# Polarized Raman spectroscopy with differing angles of laser incidence on single-layer graphene

**DOI:** 10.1186/s11671-015-0743-4

**Published:** 2015-02-06

**Authors:** Gaeun Heo, Yong Seung Kim, Seung-Hyun Chun, Maeng-Je Seong

**Affiliations:** Department of Physics, Chung-Ang University, Seoul, 156-756 Republic of Korea; Department of Physics, Sejong University, Seoul, 143-747 Republic of Korea

**Keywords:** Graphene, Polarized Raman, Oblique incidence, Raman selection rule

## Abstract

Chemical vapor deposition (CVD)-grown single-layer graphene samples, transferred onto a transmission electron microscope (TEM) grid and onto a quartz plate, were studied using polarized Raman spectroscopy with differing angles of laser incidence (*θ*). Two different polarization configurations are used. In an *in-plane configuration*, the polarization direction of both incident and scattered light is parallel to the graphene plane. In an *out-of-plane configuration*, the angle between the polarization vector and the graphene plane is the same as the angle of laser incidence (*θ*). The normalized Raman intensity of the G-band measured in the *out-of-plane configuration*, with respect to that in the *in-plane configuration*, was analyzed as a function of *θ*. The normalized Raman intensity showed approximately cos^2^*θ*-dependence up to *θ* = 70°, which can be explained by the fact that only the electric field component of the incident and the scattered photon in the *out-of-plane configuration* projected onto the graphene plane can contribute to the Raman scattering process because of the perfect confinement of the electrons to the graphene plane.

## Background

Graphene, an almost perfect two-dimensional crystal, has been intensively investigated for the last decade, since its discovery in 2004 [1,2], due to its remarkable physical properties such as high thermal conductivity, high mobility, room-temperature quantum Hall effect, outstanding flexibility, and tunable bandgap [3-11].

Raman spectroscopy has played an important role in studying graphene [12,13]. A typical Raman spectrum of graphene consists of the D-band near 1,340 cm^−1^, the G-band near 1,585 cm^−1^, and the 2D-band at approximately 2,675 cm^−1^. By analyzing both the normalized Raman intensity of the 2D-band with respect to that of the G-band and the line-shape of the 2D-band, the number of graphene layers can be accurately determined [13]. Recently, the C-mode, which is known to be sensitive to the interlayer coupling in multilayer graphene, was observed and it would, in principle, be absent from the Raman spectrum of single-layer graphene [14].

Polarizations of the incident laser and the scattered light are important in the Raman scattering on low-dimensional crystals. For a carbon nanotube (CNT), which is a quasi-one-dimensional crystal, the Raman intensities of CNT vibrational modes are maximum when the polarization directions of both the incident and the scattered light are parallel to the CNT direction whereas the Raman scattering is forbidden when the polarization directions of both the incident and the scattered light are perpendicular to the CNT direction [15]. In fact, the Raman intensities of the G-band and the radial breathing mode (RBM) of the CNT exhibited approximately *cos*^2^*α*-dependence in which *α* is the angle between the CNT axis and the polarization direction of the incident light [15]. Similar polarization anisotropies in polarized Raman scattering from CNTs were reported [16-19].

**Figure 1:**
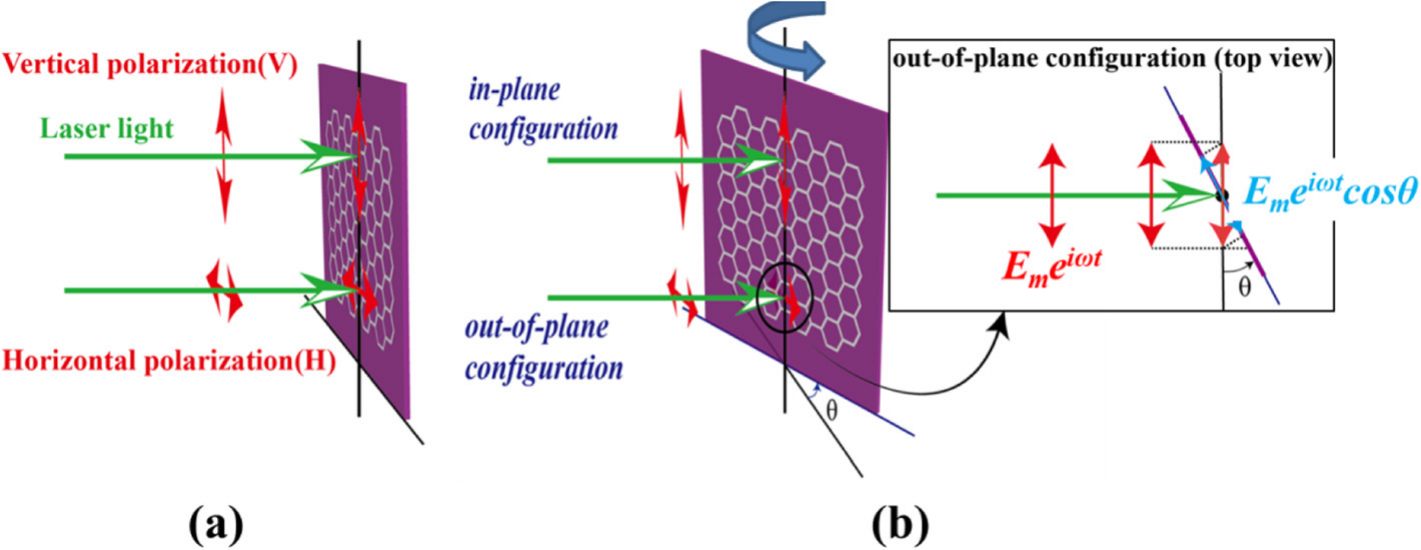
**Schematic diagram of the polarization configurations in our polarized Raman spectroscopy. (a)** normal laser incidence (*θ* = 0), where both the vertical polarization (VV) and the horizontal polarization (HH) of the laser light are parallel to the graphene plane, **(b)** oblique laser incidence (*θ* ≠ 0) where the vertical polarization is parallel to the graphene plane (*in-plane configuration*) but the horizontal polarization is not parallel to the graphene plane (*out-of-plane configuration*). The inset shows the top view of the graphene plane and the polarization vector in the *out-of-plane configuration* in an oblique laser incidence, where the polarization projected onto the graphene plane is *E*_*m*_ *e* ^*iωt*^cos*θ*.

For a two-dimensional crystal, polarized Raman spectroscopy on single-layer graphene revealed strong polarization anisotropy for the Raman intensity of the double-resonant 2D-band, whereas that of the G-band is isotropic, under the normal laser incidence in the backscattering geometry [20]. Polarization anisotropy for the Raman intensity of the 2D-band on bilayer graphene was also reported [21,22]. All the polarized Raman studies on graphene, reported to date, were performed in the backscattering geometry with the normal laser incidence, i.e., the propagation direction of the incident and the scattered light is perpendicular to the plane of the graphene. For the normal laser incidence in the backscattering geometry, the electric field vector of the incident and the scattered light is always fully contained in the graphene plane, irrespective of the polarization direction of the light, as schematically illustrated in Figure 1a. However, when the laser incidence is not normal but oblique with the incident angle of *θ*, the electric field vector of the incident light can be fully contained in the graphene plane (*in-plane configuration*) or it can make an angle *θ* (*out-of-plane configuration*), depending on the polarization direction of the incident light, as shown in Figure 1b.

In this work, we investigated polarization anisotropy of the G-band and the 2D-band of single-layer graphene for oblique laser incidence with differing angles of laser incidence for the first time. The normalized Raman intensity of the 2D-band measured in the *out-of-plane configuration*, with respect to that in the *in-plane configuration*, was analyzed as a function of *θ*. The normalized Raman intensity showed approximately cos^2^*θ*-dependence up to *θ* = 70°.

## Methods

Single-layer graphene films were grown on 25-μm thick Cu foils (Alfa Aesar, 99.8%) by a commercial plasma-enhanced chemical vapor deposition (CVD) system (Atech System, Incheon, Korea) at 830°C using methane. Details can be found in ref. [23].

For Raman spectroscopy, the synthesized graphene films were transferred either onto transmission electron microscope (TEM) grids or onto quartz substrates by etching the Cu foil in an aqueous solution of FeCl_3_. Prior to wet-etching, the surface of graphene/Cu was spin-coated with poly-methyl methacrylate (PMMA: 950 A2), followed by baking at 150°C for 3 min. Once Cu foil was dissolved completely, the PMMA/graphene membrane was washed with deionized water and placed on a TEM grid or a quartz substrate. Finally the PMMA film was removed by acetone.

Raman spectra were measured at room temperature by using a 532-nm line from a frequency-doubled Nd:YAG laser (CL532-200-S; Crystalaser, Reno, USA) as the excitation light source. Scattered light from the samples was analyzed through a single grating spectrometer (1,200 grooves/mm) with a focal length of 50 cm (SP-2500i; Princeton Instruments, Trenton, USA) and detected with a liquid-nitrogen-cooled silicon CCD detector (Princeton Instruments, Spec-10). The laser spot size on the sample was approximately 100 μm, and the spectral resolution was approximately 1 cm^−1^.

Two different Raman scattering geometries were used in this work. The backscattering geometry was used for the single-layer graphene on a TEM grid but the forward-scattering geometry for that on a quartz plate. For both scattering geometries, the vertical direction is assigned to the polarization direction of the incident light when its electric field is fully contained in the graphene plane whereas the horizontal direction is assigned to that when its electric field made the same angle *θ* with the graphene plane as the laser incident angle *θ*. Thus, the *in-plane configuration* and the *out-of-plane configuration* can be denoted by (VV) and (HH), respectively. Polarized Raman spectra were measured in both (VV) and (HH) configurations for each laser incident angle *θ* ranging from −70° to 70°.

## Results and discussion

**Figure 2:**
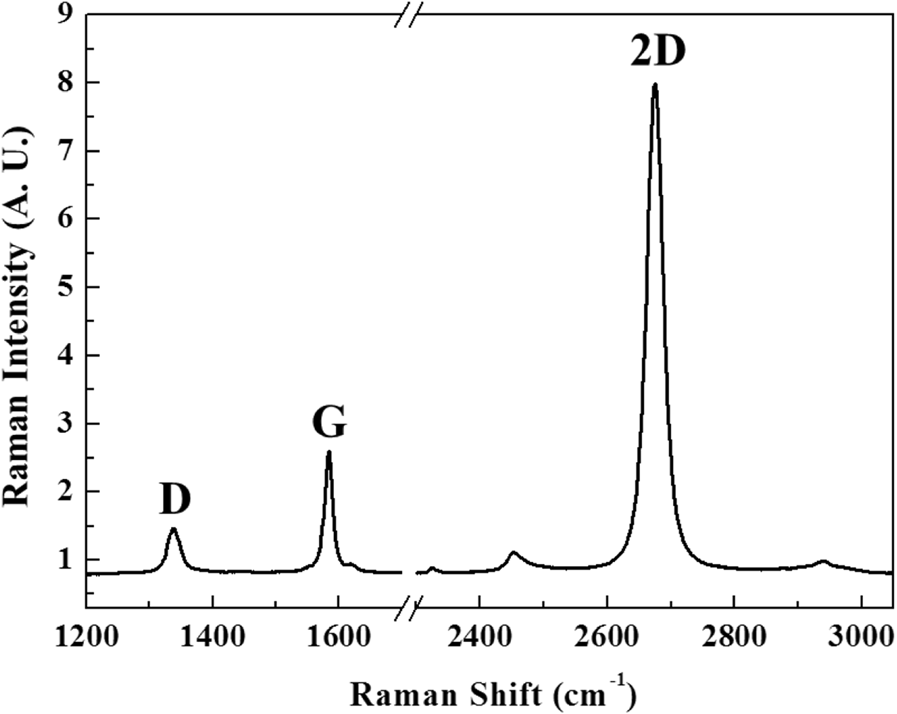
**Raman spectrum of the monolayer graphene excited with a 532**-**nm laser.** The 2D-band Raman intensity is four times stronger than that of the G-band.

Raman spectrum of the CVD-grown graphene sample used in this work is shown in Figure 2. The 2D-band Raman intensity is four times stronger than that of the G-band, indicating that the sample is single-layer graphene.

**Figure 3:**
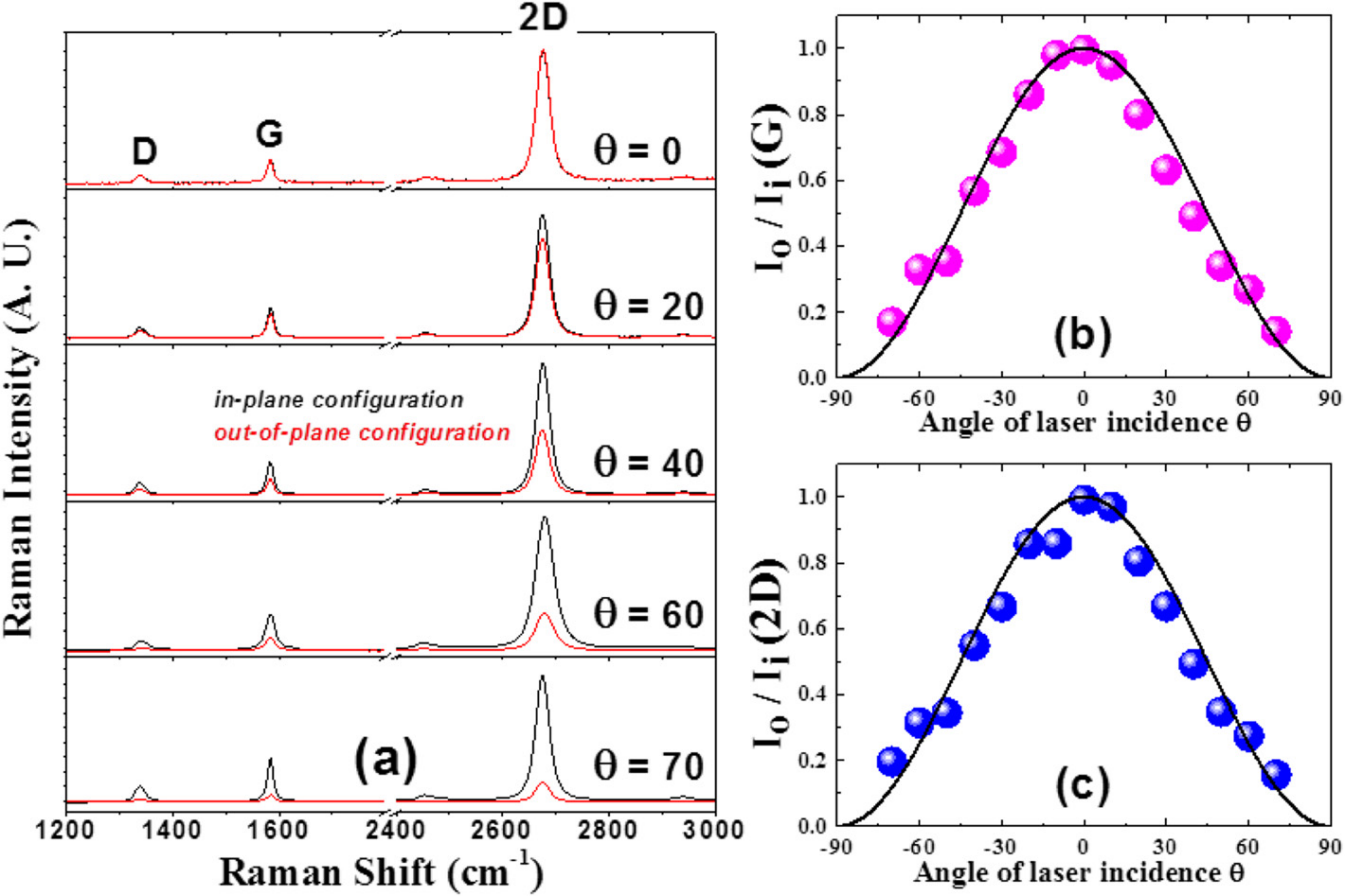
**Polarized Raman spectra of a monolayer graphene on a TEM grid in the backscattering geometry. (a)** Polarized Raman spectra of a monolayer graphene on a TEM grid in the backscattering geometry with differing angles (*θ*) of the laser incidence. Excitation laser wavelength was 532 nm. The black curves are Raman spectra measured with *in-plane configuration* and the red ones measured with *out-of-plane configuration*. **(b**, **c)** Normalized Raman intensity of the G-band **(b)** and the 2D-band **(c)** in *out-of-plane configuration* with respect to that in *in-plane configuration* as a function of laser incidence angle, respectively. The black lines represent cos^2^ *θ*.

Figure 3a shows the Raman spectra for different angles of laser incidence onto the single-layer graphene on a TEM grid in the backscattering geometry, where the black lines and the red lines correspond to the Raman spectra taken in (VV) and (HH) polarization configurations, respectively. In the (HH) polarization configuration, the entire electric field vector of the incident light cannot interact with the electrons on the graphene whereas it can interact with them in the (VV) polarization configuration, as shown in Figure 1b. In fact, only the projected component of the electric field vector of the incident light onto the graphene plane can effectively contribute to Raman scattering process as illustrated in Figure 1b. This is exactly the same situation as the polarized Raman scattering on single isolated CNT, where the Raman intensities of the G-band and the RBM of the CNT exhibited approximately cos^2^*α*-dependence in which *α* is the angle between the CNT axis and the polarization direction of the incident light [15]. Thus, the normalized Raman intensities of the G-band and the 2D-band measured in *out-of-plane configuration* with respect to that measured in *in-plane configuration*, $\frac{I_o\left(\theta \right)}{I_i\left(\theta \right)}\kern0.5em (G)$ and $\frac{I_o\left(\theta \right)}{I_i\left(\theta \right)}\kern0.5em \left(2\mathrm{D}\right)$, respectively, were expected to exhibit cos^2^*θ*-dependence. Experimental data, shown in Figure 3b,c, for the G-band $\frac{I_o\left(\theta \right)}{I_i\left(\theta \right)}\kern0.5em (G)$ and the 2D-band $\frac{I_o\left(\theta \right)}{I_i\left(\theta \right)}\kern0.5em \left(2\mathrm{D}\right)$ as a function of *θ*, agreed quite well with cos^2^*θ*-dependence (the black solid line) as expected.

**Figure 4:**
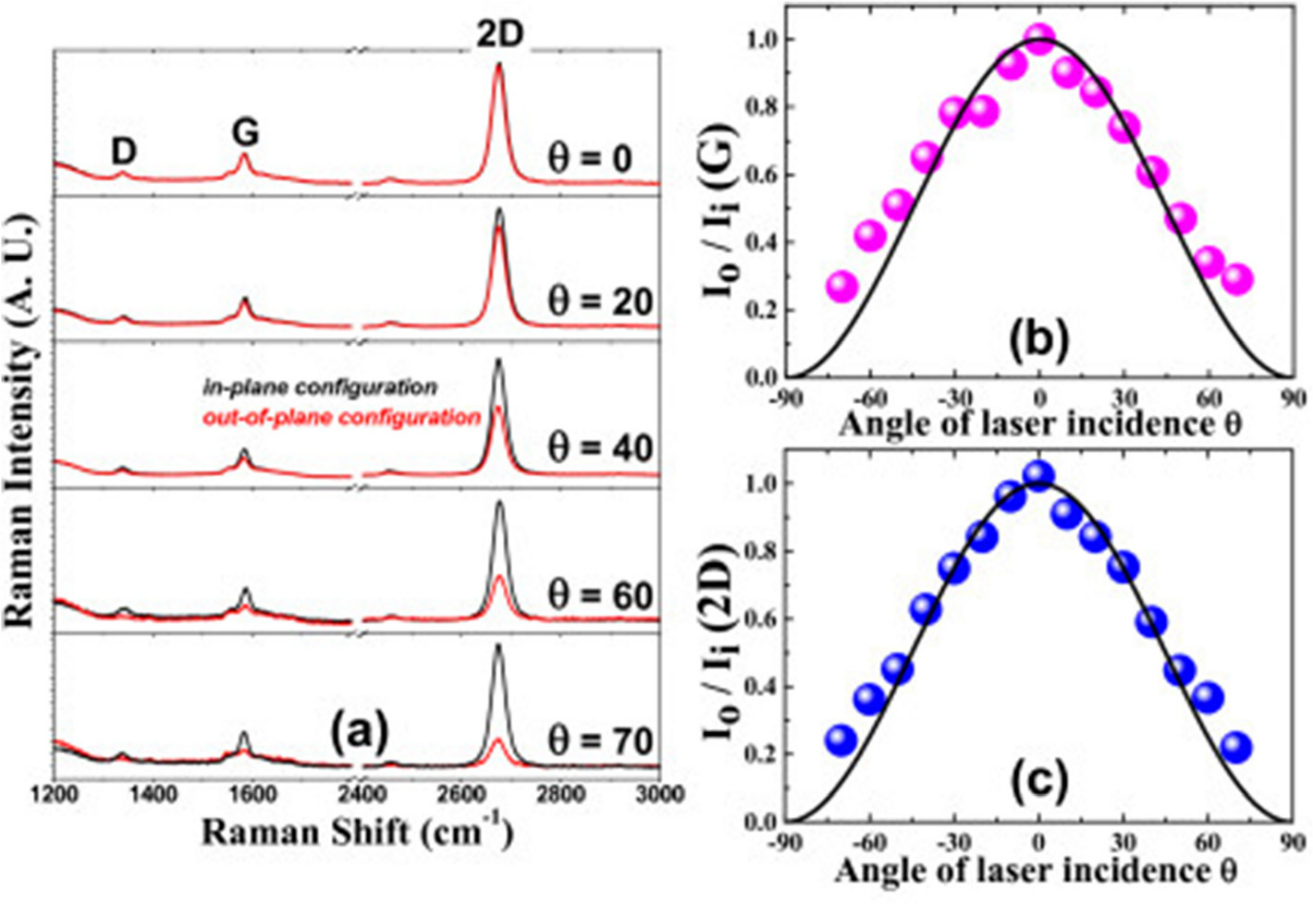
**Polarized Raman spectra of a monolayer graphene on a quartz plate. (a)** Polarized Raman spectra of a monolayer graphene on a quartz plate in the forward-scattering geometry with differing angles (*θ*) of the laser incidence. Excitation laser wavelength was 532 nm. The black curves are Raman spectra measured with *in-plane configuration* and the red ones measured with *out-of-plane configuration*. **(b**, **c)** Normalized Raman intensity of the G-band **(b)** and the 2D-band **(c)** in *out-of-plane configuration* with respect to that in *in-plane configuration* as a function of laser incidence angle, respectively. The black lines represent cos^2^ *θ*.

Figure 4a shows the Raman spectra for different angles of laser incidence onto the single-layer graphene on a quartz plate in the forward-scattering geometry, where the black lines and the red lines correspond to the Raman spectra taken in (VV) and (HH) polarization configurations, respectively. The polarization anisotropy shown in Figure 4a is almost the same as that in Figure 3a. The normalized Raman intensity, $\frac{I_o\left(\theta \right)}{I_i\left(\theta \right)}$, of the G-band and the 2D-band exhibited cos^2^*θ*-dependence as shown in Figure 4b,c, respectively.

**Figure 5:**
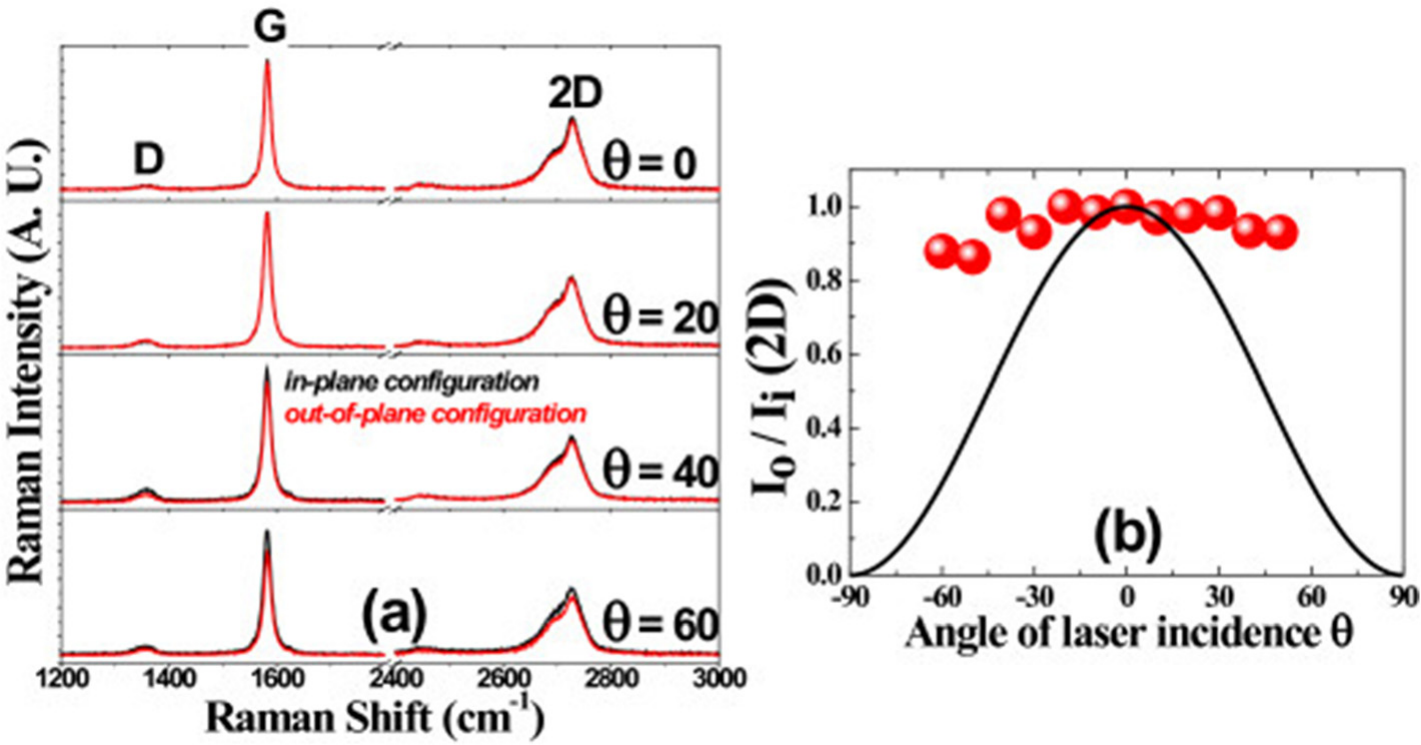
**Polarized Raman spectra of a bulk graphite in the backscattering geometry. (a)** Polarized Raman spectra of a bulk graphite in the backscattering geometry with differing angles (*θ*) of the laser incidence. **(b)** Normalized Raman intensity of the 2D-band in *out-of-plane configuration* with respect to that in *in-plane configuration* as a function of laser incidence angle. The black line represents cos^2^ *θ*.

In order to double-check that the observed polarization anisotropy in Figures 3 and 4 originates from the two-dimensional nature of graphene, we performed the same measurements on bulk graphite for which such polarization anisotropy was not expected. Indeed, the normalized Raman intensity, $\frac{I_o\left(\theta \right)}{I_i\left(\theta \right)}$, of the G-band and the 2D-band showed almost isotropic behavior as shown in Figure 5.

## Conclusions

We investigated polarization anisotropy of the G-band and the 2D-band of single-layer graphene for oblique laser incidence with differing angles of laser incidence for the first time. The normalized Raman intensity of the 2D-band measured in the *out-of-plane configuration* with respect to that in the *in-plane configuration* was analyzed as a function of the laser incident angle *θ*. The normalized Raman intensity $\frac{I_o\left(\theta \right)}{I_i\left(\theta \right)}$ of the G-band and the 2D-band showed approximately cos^2^*θ*-dependence up to *θ* = 70°, which is the direct consequence of the two-dimensional nature of graphene.

## Abbreviations

CCD: charge coupled device
CNT: carbon nanotube
CVD: chemical vapor deposition
PMMA: poly-methyl methacrylate
RBM: radial breathing mode
TEM: transmission electron microscope
